# Quality Matters: Influences of Citrus Flush Physicochemical Characteristics on Population Dynamics of the Asian Citrus Psyllid (Hemiptera: Liviidae)

**DOI:** 10.1371/journal.pone.0168997

**Published:** 2016-12-28

**Authors:** Mamoudou Sétamou, Catherine R. Simpson, Olufemi J. Alabi, Shad D. Nelson, Srilakshmi Telagamsetty, John L. Jifon

**Affiliations:** 1 Department of Agriculture, Agribusiness and Environmental Sciences, Texas A&M University-Kingsville Citrus Center, Weslaco, Texas, United States of America; 2 Department of Plant Pathology & Microbiology, Texas A&M AgriLife Research and Extension Center, Weslaco, Texas, United States of America; 3 Department of Horticulture, Texas A&M AgriLife Research and Extension Center, Weslaco, Texas, United States of America; Instituto de Biologia Molecular y Celular de Plantas, SPAIN

## Abstract

Studies were conducted to relate the influence of the physical characteristics, leaf nutrient content and phloem sap amino acid concentration of citrus flush shoots on the densities of various *Diaphorina citri* life stages. Adult *D*. *citri* preferentially selected young shoots for feeding and numbers of *D*. *citri* immatures were positively correlated with flush shoot softness. Young flush shoots had higher concentrations of macro and micro nutrients relative to mature ones and this was associated with higher densities of all *D*. *citri* life stages. All *D*. *citri* life stages were positively correlated with higher nitrogen-carbon (N:C), nitrogen:sulfur (N:S) and nitrogen:calcium (N:Ca) ratios in leaf tissue, while densities of adults were negatively related to calcium, manganese and boron levels. Concentrations of total and essential amino acids were highest in phloem sap of young expanding flush shoots in both grapefruit and lemon, but dramatically declined as flush shoots matured. The sulfur-containing amino acids cystine, methionine and taurine occurred only in younger flush shoots. In contrast, cystathionine was only present in phloem sap of mature shoots. These results clearly indicate that young citrus flush shoots are a nutritionally richer diet relative to mature shoots, thus explaining their preference by *D*. *citri* for feeding and reproduction. Conversely, tissue hardness and the lower nutritional quality of mature flush shoots may limit oviposition and immature development. The data suggest that both physical characteristics and nutritional composition of flush shoots and their phloem sap are important factors regulating host colonization and behavior of *D*. *citri*, and this interaction can impact the dynamics and spread of HLB in citrus groves.

## Introduction

The Asian citrus psyllid, *Diaphorina citri* Kuwayama, 1908 (Hemiptera: Liviidae) is the vector of the phloem-inhabiting bacterial pathogen *Candidatus* Liberibacter asiaticus (CLas), the primary causal agent of Huanglongbing (HLB) or citrus greening disease in the U.S. [1–4]. Both ACP and HLB have invaded the U.S. during the last decade. The disease was first detected in Florida in 2005 [5,6] and subsequently in California and Texas in 2012 [5,7]. In Florida, HLB has caused substantial economic losses totaling more than $4.5 billion from 2006–2012, besides the loss of over 8,000 jobs [8]. HLB is currently considered the most devastating disease threatening the viability of global citrus production, and for which there is no known cure [2]. Timely and effective control of its vector, *D*. *citri* is a key management strategy for reducing the incidence and spread of HLB [9].

*D*. *citri* is an oligophagous phloem-feeding insect that develops on rutaceous host plants and reproduces exclusively on juvenile foliage (flush shoots) of its hosts [5,10]. Due to the restriction of reproduction on juvenile and expanding flush shoots, it is very likely that flush shoot characteristics and nutritional quality may dictate the reproductive potential of *D*. *citri* including mating, egg maturation and successful immature development. Flush shoots of citrus and other rutaceous host plants of *D*. *citri* often exhibit distinct morphological changes during growth and development [11]. Such ontogenetic changes are typically associated with variations in physicochemical properties such as texture and chemical composition of leaf tissue and phloem sap of the flush shoots [11]. Therefore, gaining an understanding of *D*. *citri* life cycle in relation to ontogenetic changes in flush shoot characteristics can shed light on the nutritional requirements of *D*. *citri* and elucidate the factors governing its population fluctuations and dispersal behavior.

The spread of vector-transmitted pathogens in plants depends not only on the ability of the vector to acquire and transmit the pathogen, but also on its mobility [12] and its overall fitness. The reproductive potential and abundance of herbivorous insects are dependent upon their host plant’s nutritional quality which are in turn determined by ontogenetic changes in plant physicochemical characteristics such as anatomical and tissue chemical (e.g. allelochemical and nutrient) composition [13,14]. Nutritional ecology studies of many phytophagous insects have demonstrated the importance of nitrogenous compounds on their survival, growth, development and reproductive parameters [13,15–18]. For phloem-feeding insects, amino acids constitute the main nitrogen source in phloem sap [13,19]. Phloem sap of mature leaves of plants is generally rich in carbohydrates but poor in amino acids and possibly also micronutrients [13,19,20]. As a consequence, many phloem feeders ingest vast quantities of carbohydrates in excess of their dietary needs in order to meet their protein requirements which explains the sugary nature of their excreted honeydew [20]. While feeding on young shoots of their host plants, *D*. *citri* nymphs secrete copious amounts of a sugary tube-like substance covered by white waxy materials [21]. ACP adult females feeding on young shoots also produce white excretory substances, but males produce clear sticky droplets [22]. Interestingly, these secretions are seldom seen or are less abundant when adults are feeding on mature and fully developed leaves and stems which would suggest marked differences in phloem composition of young versus matured shoots.

The chemical composition of phloem sap varies with plant species and cultivars, edaphic conditions, time of day, season, as well as growth stage [16,20,23]. It is therefore possible that the suitability of phloem sap as a nutrient source may fluctuate accordingly. Such changes in phloem sap chemical composition together with changes in physical characteristics of flush shoots from emergence to maturity may explain their differential colonization by *D*. *citri* and suitability for immature development.

In citrus, young flush shoots are mostly produced during the discrete flush cycles occurring between post-winter dormancy and early fall in most citrus producing states in the U.S. which often correlate with peaks of ACP density [24]. It has been shown that *D*. *citri* population density on host plants is tightly linked to the presence of young flush shoots on such plants [10,24,25]. Therefore, it is plausible that adult *D*. *citri* feeding on plant parts that contain the highest nutrient concentrations are more reproductively active, leading to an increase their population densities. However, there is a dearth of information on the key plant factors that affect *D*. *citri* host suitability. Such knowledge is needed to develop efficient alternative management strategies for *D*. *citri* based on host plant parameters. The current nutritional ecology study was conducted to better understand how citrus flush shoot characteristics regulate *D*. *citri* host preference/colonization and population densities. Specific objectives were (i) to record and compare the population densities of *D*. *citri* life stages on flush shoots of various growth stages for grapefruit and lemon; (ii) to document changes in textural characteristics (firmness or physical hardness) of flush shoots during ontogenesis; (iii) to analyze and quantify leaf tissue nutrient contents and free amino acid concentrations in phloem sap of citrus flush shoots at various growth stages; and (iv) to assess the relationships between flush shoot physical/nutritional characteristics and *D*. *citri* population densities recorded on such shoots.

## Materials and Methods

### Citrus cultivars and flush cycles

Two citrus cultivars, grapefruit (*Citrus* × *paradisi* Macfad., var. ‘Rio Red’) and lemon (*C*. × *limon* [L.] Burm., var. ‘Meyer’) were used in the study. Five mature (15 year-old bearing) trees per cultivar were selected from an assorted varietal block planted in a calcareous-clay soil with pH of 7.5–7.8 at the Texas A&M University-Kingsville Citrus Center, Weslaco, Texas. The block has been managed following standard commercial grove care practices including regular flood irrigation, fertilization and conventional pest and disease management programs. However, the experimental block was not sprayed with insecticides during the entire duration of study from April 2014 to June 2015. Trees were fertilized on 4 February, 2014 and again on 26 February, 2015 with urea (112 kg N/ha). In addition, a foliar spray with a complete fertilizer (Foligro Action: 2% N, 17% P2O5, 17% K2O, 0.1%Fe, 0.08% Mn, 0.0005% Mo and 0.15% Zn; Wilbur-Ellis, Yakima, WA) was made on 15 April, 2014 and on 20 August, 2014 at a rate of 9.35 L/ha in a total of spray volume of 1,871 L/ha. In addition, the experimental trees were confirmed to be HLB-negative by qPCR using standardized assays [7,26]. Presence of young flush shoots on the experimental trees was monitored and recorded following the five-stage categories proposed by Arredondo [27] which is based on their physical characteristics including color, hardness and architecture. The categories were: SI = feather flush or young, soft, newly-unfurled, yellow-green leaves and with < 1 cm stem length (from base of stem to the point of attachment of first leaf) and generally one-week old or less; SII = expanding light green and soft leaves with stem 1–2.5 cm long, typically 1–2 weeks old; SIII = fully expanded, soft and light-green leaves with stem <5 cm long, typically 2–3 weeks old; SIV = mature, firm and green leaves with stem > 5 cm long, typically 3–4 weeks old; and SV = fully matured, hard, and dark green leaves with stem > 5 cm long, generally more than 4 weeks old [27]. Although *D*. *citri* eggs are mostly deposited on unfurled flush shoots, nymphs are routinely found feeding along the pedicel of leaves and the stem of the expanding flush shoot. Hence, the SII and SIII stages on which *D*. *citri* nymphs are routinely recorded were grouped together as “young flush shoots” while fully expanded SIV and SV stages were grouped and referred to as “mature flush stages” for the purpose of this study.

### Measurement of flush shoot hardness

Flush shoot hardness was measured during the fall (September-October) flush cycle. Young and mature flush shoots were collected from experimental citrus trees a week after a heavy rain event (~100 mm precipitation) on 15 September 2014. Shoots were excised from their point of attachment to the main stem early in the morning, wrapped in moist tissue paper and immediately transported on ice to the laboratory for processing. A cohort of 40 flush shoots (ten shoots per growth stage for a total of 20 shoots per cultivar) was individually weighed using an analytical balance (Sartorius CP124S, Data Weighing Systems, Elk Grove, IL). Subsequently, 10 young and 10 mature flush shoots for each of the two cultivars were used for hardness measurements. Flush shoot hardness readings were made at the base of the petiole of the first leaf using a digital penetrometer (Model Force One FDI Series, Wagner Instruments, Greenwich, CT) fitted with a flat-tipped ‘minuten’ probe of 0.254 mm diameter. Firmness or hardness readings were expressed as kilogram-force (kgf) or the force required for the penetrometer pin to puncture the flush tissue.

### Phloem sap analysis for free amino acid content

Approximately 20 g of young and mature flush shoots were collected from each of the two cultivars for phloem sap extraction and nutrient analysis during the major flush cycles of summer (June), fall (September) 2014 and spring (March 2015). In preliminary trials, it was determined that it took approximately 15–20 young and four to six mature shoots to yield comparative amounts of phloem sap. Therefore, varying numbers of flushes were collected per flush stage due to observed discrepancies in size for each flush stage. The EDTA exudation technique [28] was used for phloem sap extraction since it has been shown to be capable of generating good quality phloem sap for subsequent analysis of proteins, sugars, lipids, RNA, viruses and metabolites in many herbaceous or woody plant species [29]. Briefly, flush shoots were excised at the point of attachment to the main twig using sterilized pruning shears and immediately immersed into 30 mL of 20 mM EDTA solution in plastic vials. The vials were then covered with moist paper towels and transported on dry ice to the laboratory to maintain sample integrity. Samples were agitated at 100 rpm on a table shaker (Innova 2300 Platform Shaker, New Brunswick Scientific, Enfield, CT) in a dark, temperature (21°C) controlled room for 3 hrs. The leaves were retrieved from the EDTA solution and the resulting EDTA-phloem sap extracts were transferred into sterile 50 mL centrifuge tubes and stored at -80°C until further processing. Prior to free amino acid analysis, tubes containing the frozen phloem sap solutions were retrieved, uncapped, and freeze-dried for 96 hrs. using a benchtop lyophilizer (Model: BT48, Millrock Technology, Kingston, NY). The dry weight of phloem exudate in each tube was recorded and samples were again placed in the -80°C freezer until further analysis. Free amino acid analysis was conducted on the dried phloem exudates at the University of Missouri-Columbia Experimental Station Chemical Laboratories (Columbia, MO). The freeze dried samples were re-suspended in 2 mL of 0.01N HCl and a 50 μL aliquot of each solution was analyzed for amino acids using a High-Speed Amino Acid Analyzer (Model: L-8900, Hitachi Ltd., Tokyo, Japan) and following the methods previously described by Deyl (1986), Le Boucher (1997) and Fekkes (1996) [30–32].

### Leaf tissue mineral nutrient content

For leaf tissue mineral content analysis, flush shoots were excised at the point of attachment to the main twig using sterilized pruning shears and transported on ice to the laboratory. Flush shoots were washed with a 0.1 N HCl solution, rinsed with deionized water to remove any adhering particulates, oven dried (65°C, 48 hrs.), then ground to pass a 40 μm screen using a benchtop tissue grinder mill (Cole-Parmer, Vernon Hills, IL). Mineral analyses were performed at a commercial laboratory operated by the Soil, Water and Forage Testing Laboratory (Texas A&M University, College Station, TX). Leaf samples were analyzed for nitrogen by the Kjeldahl method and for other nutrients by inductively-coupled plasma emission (ICPE) spectroscopy. Carbon content was analyzed using a CHN analyzer. Three samples were analyzed for young and mature flush stages for grapefruit and lemon during each of the major flush cycles of summer (June) and fall (September) 2014 and of spring (March 2015) for all minerals with the exception of C for which only one sample was analyzed.

### Asian citrus psyllid population monitoring

*D*. *citri* population densities were recorded fortnightly on 20 randomly selected flush shoots per tree from four of the five experimental grapefruit and lemon trees from May 2014 to May 2015. Efforts were made to select 10 young and 10 mature flush shoots during flush cycles when new flush shoots were present. In most instances however, only the mature flush shoots were present at any given time during the season. Flush shoots were carefully examined and numbers of ACP adults were first recorded per flush shoot. Thereafter, numbers of ACP nymphs were counted *in situ* on the same flush shoots using a 5X hand lens. Only the proportion of flush shoots infested with eggs were recorded because enumeration of *D*. *citri* eggs on flush shoots was too time consuming.

### Feeding preferences of *D*. *citri* on citrus flush shoots

Choice tests were conducted to investigate the feeding preferences of adult psyllids on grapefruit and lemon flush shoots. Young and mature shoots were excised from trees during the fall flush cycle on 23 September 2014 in the experimental block and immediately inserted into aquatubes containing hydroponic solution (General Hydroponics, Sebastopol, CA) to maintain their turgidity. Sétamou and Bartels [24] have previously reported higher *D*. *citri* populations during the fall flush cycle (September-October) than any other cycle during the year. A choice test was conducted to assess the preference of psyllid adults for young versus mature flush shoots for each of the two cultivars. Similarly, a host preference test was conducted using flush shoots of the same growth stage (young or mature). Thus, four separate tests were conducted under illumination (~50 μmol m^-2^ s^-1^) in the laboratory following the protocol previously described by Sétamou et al. [33]. Briefly, for each test, clear glass desiccators (Secador Techni-Dome 360 Vacuum Desiccator, Bel-Art Products, www.belart.com) with a perforated circular tray to support aquatubes were used. Eight flush shoots (four for each the two treatments being tested) were alternately placed along a diameter of the tray. A clear-plastic vial containing 10 pairs of mated *D*. *citri* adults was placed in the middle of the perforated tray. The lid of the vial was carefully removed to allow adult psyllids to freely choose their feeding sites, and the desiccator was immediately covered. For each of the four tests, there were 10 replications. Numbers of adult *D*. *citri* that settled on each flush shoot were recorded after 24 h.

### Statistical analysis

All statistical analyses, except where noted, were conducted using SAS 9.4 [34]. Flush shoot hardness data were subjected to a two-way analysis of variance to evaluate the effects of cultivar and growth stage and their interaction. When significant differences were detected (*P* < 0.05), cultivar or flush shoot mean values were separated using Tukey’s test. Various nutrient ratios including the nitrogen:carbon (N:C), nitrogen:sulfur (N:S) and the nitrogen:calcium (N:Ca) ratios of leaf samples were calculated per flush stage. Data on leaf tissue nutrient contents and amino acid concentrations of phloem sap for each flush cycle were first subjected to two-way analysis of variances with cultivar, flush stage and their interaction as independent factors. Since results of the analysis showed significant interactions between cultivar and flush stage for most dependent variables, suggesting that the effect of flush stage varied with citrus cultivar, mean data of flush stage for leaf nutrient and phloem amino acid contents were compared separately for each cultivar using Tukey’s test.

Because many leaf tissue minerals and phloem amino acid concentrations were highly correlated, a principal component analysis (PCA)–a dimension reduction technique—with a correlation matrix to standardize variables [35] was used to analyze plant nutrient contents and phloem sap amino acid concentrations as quantitative independent variables and their relationships with flush growth stage (young vs mature) and citrus cultivar (grapefruit and lemon) as classification dependent variables. The analysis was performed by transforming the data to achieve a multivariate normal distribution and then extracting principal components (PC) from all leaf tissue mineral contents and amino acid concentrations. Only PC with eigenvalues >1 and nutrients with loading values >0.7 within each PC were considered. PCA was conducted with XLSTAT in Microsoft Excel 2007/XLSTAT-Pro (Version 6.1.9, 2003, Addinsoft, Inc., Brooklyn, NY, USA).

A repeated measures analysis of variance was used to evaluate the effects of the fixed factors i.e. cultivar (grapefruit or lemon), flush shoot growth stage (young or mature), time and their interactions with one another on densities of *D*. *citri* immatures and adults with flush shoot selected within each tree used as a random factor using the PROC MIXED procedures of SAS ([36]). Least squares (LS) means of *D*. *citri* numbers over the sampling period were calculated per flush shoot growth stage for each cultivar and compared using Tukey’s range test. To better visualize *D*. *citri* populations over time on citrus flush shoots, numbers of nymphs and adults recorded over the sampling period were expressed as cumulative psyllid-days calculated using the formula:

$$psyllid-days=\sum_{i=1}^{n}([x_{i}+x_{i-1}]/2]\;*\;t_{i}),$$

where *n* is the number of sampling date from the first one on May 2, 2014, *x*_*i*_ is the mean number of psyllids per flush shoots on sampling date *x*_*i*_, and *t*_*i*_ is the number of days between two sampling dates. A log-likelihood ratio test was used to compare cumulative psyllid-days between flush growth stage and host plants. Correlation analyses were used to determine the relationships between leaf nutrient contents and phloem sap amino acid concentrations with cumulative psyllid-days for each flush shoot sampling period. The percentage of young and mature flush shoots infested by *D*. *citri* life stages was calculated per tree for each sampling period for both cultivars. A repeated measures analysis was similarly used for the percentage of flush shoots infested by *D*. *citri* with the exception that only host cultivar, sampling date and their interaction were used as fixed factors, while each tree within a cultivar was a random variable. LS means of percent flush shoots infested by *D*. *citri* were calculated and compared between host plants using Tukey’s test. Percent flush shoot infested by *D*. *citri* was arcsine-transformed and *D*. *citri* densities were log (x+1)-transformed to homogenize variances and normalize data before analysis.

A log-likelihood test was run to evaluate the feeding preference of *D*. *citri* recovered from each flush shoot treatment for the four choice tests [37].

## Results

### Physical characteristics of young and mature flush shoots

The mean fresh weight of flush shoots varied significantly between the two cultivars (*F* = 48.84; df = 1, 36; *P* < 0.0001) such that fresh grapefruit flush shoots (0.84 ± 0.09 g/flush for young and 6.72 ± 0.69 g/flush for mature) weighed more than fresh lemon shoots (0.38 ± 0.05 g/flush for young and 4.35 ± 0.23 g/flush for mature) for the same growth stage. Similarly, the larger mature flush shoots weighed significantly more than the young expanding ones (*F* = 23.97; df = 1, 36; *P* < 0.0001). No cultivar by flush stage interaction was observed for flush shoot weight (*F* = 2.21; df = 1, 36; *P* = 0.29).

The hardness of citrus flush shoots also varied with cultivar (*F* = 8.84; df = 1, 36; *P* = 0.005) and flush shoot developmental stage (*F* = 16.42; df = 1, 36; *P* <0.0001). The interaction between host plant and flush growth stage significantly affected flush shoot hardness (*F* = 12.37; df = 1, 36; *P* = 0.003). In both cultivars, mature flush shoots (0.70 ± 0.05 kgf for grapefruit and 0.39 ± 0.04 kgf for lemon) were substantially harder than young ones (0.07 ± 0.01 kgf for grapefruit and 0.06 ± 0.01 kgf for lemon). Although no differences were observed in young flush shoot hardness between the two host plants, mature shoots of grapefruit were significantly harder than their lemon counterparts.

### Nutrient profile of citrus leaves

Concentrations of most individual mineral nutrients analyzed during the three major flush cycles of summer (July) and fall (September) 2014 and spring (March) 2015 were significantly affected by host plant (*F* > 8.84; df = 1, 40; *P* < 0.005), flush shoot growth stage (*F* > 36.46; df = 1, 40; P <0.0001), and by the interaction between host plant and flush growth stage (*F* > 36.46; df = 1, 40; P <0.0001) (Table 1). Because of the significant interactions between flush shoot growth stage and host plant for most minerals, the effect of flush growth stage on leaf nutrient content was studied separately for each host plant. Young flush shoots had higher concentrations of nitrogen, phosphorus and sodium than mature shoots during all flush cycles, while the reverse was true for calcium, magnesium and boron. The zinc content of leaf tissue was higher in young flush shoots compared to mature ones during the spring and fall flush cycles, but no significant differences were observed for this element during the summer flush cycle. Similarly, the N:C, N:S and N:Ca ratios were higher for young shoots relative to mature ones (Table 1). The concentration of other nutrients such as K, Fe, Cu and S were generally not affected by the flush growth stage (Table 1).

**Table 1:** Nutrient profiles of young and mature grapefruit and lemon flush shoots collected at different seasons in 2014–2015.

	Grapefruit								Lemon							
	Jul-14		Sep-14		Dec-14*		Mar-15		Jul-14		Sep-14		Dec-14*		Mar-15	
mg·g^-1^	Y	M	Y	M	Y	M	Y	M	Y	M	Y	M	Y	M	Y	M
Nitrogen	37.4a	29.1b	34.9a	25.9b	-	23.1	44.3a	22.3b	42.7a	27.3b	52.1a	28.0b	-	29.0	51.5a	22.1b
Phosphorus	4.56a	1.71b	5.5a	2.3b	-	2.06	6.20a	1.6b	4.2a	1.7b	6.2a	1.7b	-	2.45	6.9a	1.4b
Potassium	18.1a	14.20a	17.3a	13.8a	-	12.7	16.6a	12.5b	17.4a	9.1b	18.4a	9.1a	-	13.14	19.7a	9.52b
Calcium	6.76b	51.67a	8.2b	45.1a	-	54.2	9.04b	40.1a	10.6b	65.9a	6.8b	41.0a	-	56.8	10.2b	50.7a
Magnesium	2.36b	3.33a	2.8b	4.4a	-	3.83	2.18b	3.3a	2.8	3.9a	2.9a	2.2a	-	2.72	2.44a	2.62a
Sodium	1.39a	0.61b	0.65b	0.44b	-	0.46	1.6a	1.2a	1.91a	0.30b	1.5a	0.28b	-	0.11	2.26a	1.25b
Zinc	0.01a	0.01a	0.02a	0.01b	-	0.013	0.024a	0.009b	0.02a	0.01a	0.03a	0.01b	-	0.012	0.03a	0.01b
Iron	0.02b	0.07a	0.03a	0.04a	-	0.033	0.029a	0.007b	0.03b	0.06a	0.03a	0.12a	-	0.034	0.02b	0.05a
Copper	0.005a	0.006a	0.01a	0.01a	-	0.006	0.009a	0.007a	0.006a	0.003b	0.01a	0.01a	-	0.007	0.01a	0.004b
Manganese	0.01a	0.03a	0.01b	0.02a	-	0.032	0.01a	0.02a	0.017b	0.036a	0.02a	0.02a	-	0.029	0.02a	0.03a
Sulfur	2.59a	3.34a	2.51a	3.27a	-	3.32	2.6a	2.4a	2.83b	3.92a	2.8a	2.6a	-	3.70	3.0a	2.94a
Boron	0.03b	0.13a	0.03b	0.12a	-	0.15	0.02b	0.13a	0.03b	0.12a	0.03b	0.01a	-	0.13	0.026b	0.14b
Carbon**	45.5	41.1	46.9	40.9	-	41.3	47.1	41.2	43.3	41.8	45.2	41.7	-	41.2	45.7	41.3
N:C ratio	0.82	0.71	0.74	0.63	-	0.56	0.94	0.54	0.99	0.65	1.15	0.67	-	0.70	1.13	0.54
N:S ratio	14.4a	8.7b	13.9a	7.9b	-	6.95	17.0a	8.6b	15.1a	7.0b	18.6a	10.8b	-	7.84	17.2a	7.5b
N:Ca ratio	5.5a	0.6b	4.3a	0.6b	-	0.4	4.9a	0.6b	4.0a	0.4b	7.7a	0.7b	-	0.5	5.0a	0.4b

* Young flush shoots not present at this time.

** Only one composite sample was analyzed for carbon per flush stage for each host plant.

Means followed by the same letter for each sampling period per cultivar are not significantly different (P > 0.05) using the Tukey’s mean comparison test.

The young flush shoots of lemon had higher concentrations of N and Na relative to their grapefruit counterparts, whereas Ca levels in leaf tissue were significantly higher in mature grapefruit flush shoots as compared to mature lemon flush shoots (Table 1).

**Fig 1:**
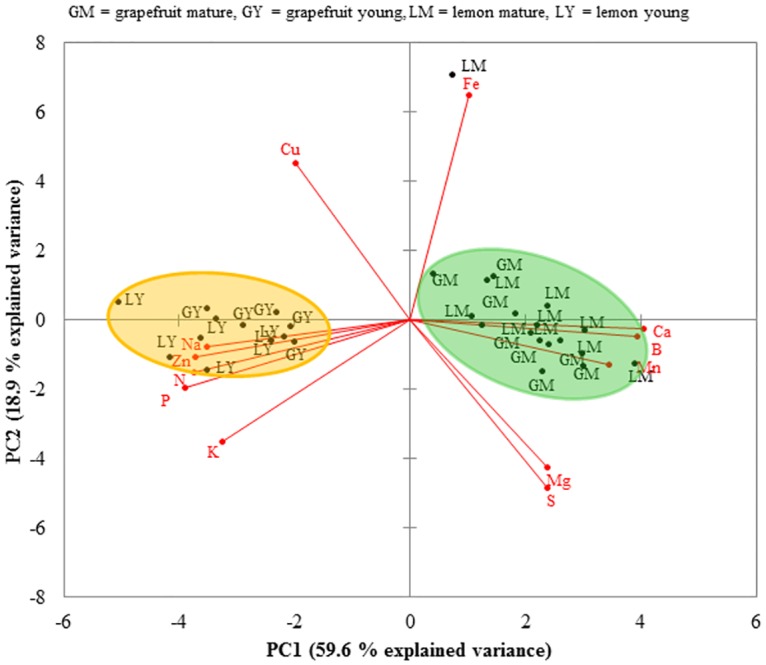
Effects of citrus flush shoot growth stage on leaf tissue mineral nutrient compositions. The analysis shows discrimination between mature and young flush shoots of both grapefruit and lemon. The explained variance of both principal component 1 (PC1) and PC2 are in parentheses (59.6% and 18.9%, respectively).

Principal component analysis of nutrient data revealed that the first two principal components each had eigenvalues >1 and explained 78.5% of the total variation (PC1 = 59.6% and PC2 = 18.9%) in leaf tissue nutrient data. All minerals with the exception of Fe and S were significantly correlated with the first principal component (PC1). The scatter plot generated using the loadings of flush stages on PC1 and PC2 clearly separated young from mature flush shoots with each flush stage clustering along PC1 (Fig 1), indicating significant differences between young and mature shoots in their nutrient profiles. PC1 increased with increasing B, Ca, Mg and Mn levels in leaf tissue, while it decreased with increasing leaf N, P, K, Cu, Na and Zn contents.

Furthermore, PC1 most strongly correlated with N and P (negatively) and with B and Ca (positively) with each nutrient explaining 80% or more of the component variance, suggesting that flush shoots with high N and P levels in leaf tissue would tend to have lower Ca and B and vice versa. Thus, PC1 can be viewed as a measure of leaf maturity and nutritional quality for psyllid immature development. The second principal component (PC2) increased with increasing Fe, while it decreased with increasing S content in leaf tissue, indicating that flush shoots with higher Fe level tend to have lower S content. Young shoots of grapefruit and lemon tended to separate along the second principal component, while mature flush shoots of the two citrus species clustered together and were difficult to separate from one another along PC2.

### Free amino acids in phloem sap

Biochemical analyses of phloem sap collected from young and mature flush shoots of grapefruit and lemon revealed the presence of both proteinogenic and non-proteinogenic amino acids. All 21 proteinogenic amino acids known to occur in eukaryotes were present with the exception of selenocysteine. In addition, cysteine was only present in the form of cystine (a disulfide-bonded twin cysteine). Concentrations of total and essential (Table 2) as well as individual free amino acids varied widely relative to one another and to the flush growth stage, season and citrus host plant (Table 2, S1 Table). In both host plants, proteinogenic amino acids represented 79–84% of total concentration of amino acids detected and did not vary significantly with flush shoot maturity (S1 Table). Mean concentrations of total free amino acids and essential amino acids were significantly higher in the phloem sap of young flush shoots relative to the phloem sap of mature ones (Table 2). In addition, there was a strong seasonal variation in total and essential amino acid concentrations in phloem sap, with the highest and lowest amounts detected in shoots collected during the spring and fall flush cycles, respectively (Table 2).

**Table 2:** Mean (± SE) total concentrations of free amino acids (TAA), essential amino acids (EAA) and their ratio (TAA:EAA) in phloem sap of young and mature grapefruit and lemon flush shoots.

Host Plant	Sampling Period	Flush shoot stage	Total amino acids (TAA, μg/mL)	Essential amino acids (EAA, μg/mL)	TAA:EAA
Grapefruit	June 2014	Young	1,769.0 ± 124.6	187.7± 11.0	10.6
		Mature	93.2 ± 6.5	7.8 ± 0.9	8.4
	September 2014	Young	1,000.6 ± 24.9	115.6± 7.8	11.5
		Mature	121.7± 11.2	9.2 ± 1.6	7.5
	December 2014	Young	.	.	.
		Mature	198.2 ± 13.1	16.3± 3.0	8.2
	March 2015	Young	2447.3 ± 295.3	341.0 ± 26.7	11.3
		Mature	112.0 ± 9.3	7.0 ± 0.9	5.3
Lemon	June 2014	Young	1,475.8 ± 108.1	251.7 ± 20.8	17.1
		Mature	129.5 ± 7.5	10.1 ± 1.9	7.8
	September 2014	Young	971.7± 31.6	94.3 ± 10.7	9.7
		Mature	130.5± 10.4	12.9 ± 2.3	9.9
	December 2014	Young	.	.	
		Mature	167.9 ± 8.6	15.0 ± 2.2	8.9
	March 2015	Young	2,238.0 ± 214.4	198.6 ± 9.4	8.9
		Mature	171.7± 12.9	11.5 ± 1.7	6.7

**Fig 2:**
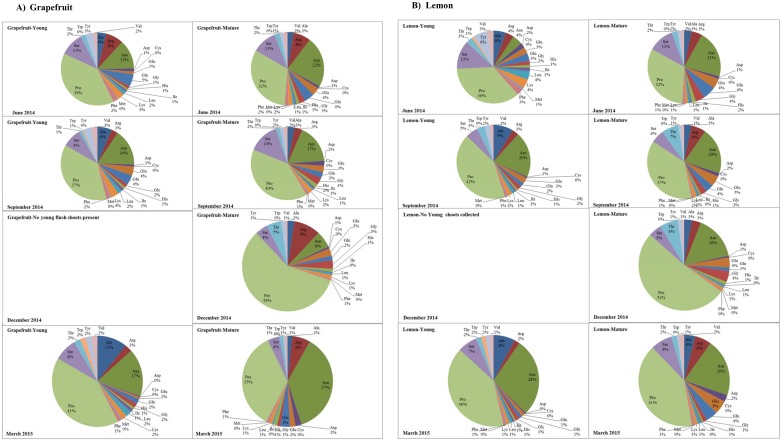
Percentage composition of proteinogenic amino acids in the phloem sap of young and mature flush shoots of grapefruit (A) and lemon (B). The analyzed phloem sap samples were collected using the EDTA exudation method at different seasons.

Total amino acid concentrations in phloem sap of young flush shoots of grapefruit and lemon were respectively 13 to 23-fold and 7 to 13-fold higher than the amounts recovered in phloem sap of their corresponding mature shoots. Similarly, the concentrations of essential amino acids in phloem sap of young shoots were 13 to 49-fold and 7 to 25-fold higher than those present in the phloem sap of mature shoots respectively for grapefruit and lemon (Table 2). In addition to quantitative variations in total and essential amino acid content observed between young and mature shoots, the relative proportions of individual amino acids also varied substantially with flush shoot growth stage (Fig 2). The free amino acids in phloem sap of both grapefruit and lemon were dominated by non-essential amino-acids (e.g. alanine, asparagine, glutamic acid, glutamine, proline, serine and γ-amino-butyric acid, Fig 2 and S1 Table). The nine essential amino acids (histidine, isoleucine, leucine, lysine, methionine, phenylalanine, threonine, tryptophan and valine) represented 5.3–11.5% and 6.7–17.1% of total amino acid concentrations in phloem sap of grapefruit and lemon, respectively. The proportions of essential amino acids in phloem sap of young shoots tended to be higher than those observed in phloem sap of mature shoots in both host plants (Table 2). The essential amino acid tryptophan and the sulfur-containing cystine were only detected in phloem sap of young shoots of both cultivars, but absent in sap of mature shoots. In addition, the sulfur-containing proteinogenic essential amino acid methionine and the non-proteinogenic amino acids α-amino-adipic acid, β-alanine, hydroxylysine, and taurine were mostly detected in phloem sap of young shoots and virtually absent in phloem sap of mature flush shoots in both grapefruit and lemon (S1 Table). In contrast, citrulline and cystathionine were absent from phloem sap of young shoots and only recorded in mature flush growth stages (S1 Table).

**Fig 3:**
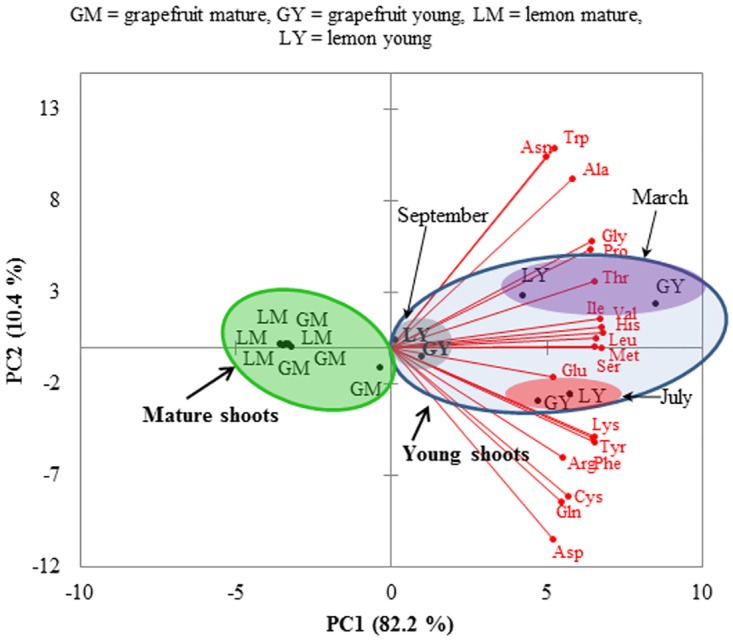
Influence of flush shoot growth stage on the composition of proteinogenic amino acids in phloem sap of grapefruit and lemon. Mature and young flush shoot stages are indicated in green and light blue shades, respectively. Within young shoot cluster, red shade depicts summer, yellow shade depicts fall collection and purple shade shows spring collection. The principal component (PC) analysis shows discrimination between mature and young shoots, and within young shoots discrimination between collection dates. The percentage of data explained by the first two components PC1 and PC2 is in parentheses (82.2 and 10.4%, respectively).

The proteinogenic amino acid profiles of phloem sap collected from young and mature shoots of grapefruit and lemon were distinct from each other when compared by principal component (PC) analysis. PC1 that explained 82.3% of the variation discriminated between phloem samples from mature and young shoots, with young shoots distributed along positive values of PC1, while mature shoots clustered together and had negative loadings on PC1 (Fig 3). All 20 proteinogenic amino acids had positive and significant loadings on PC1. PC2 explained 10.4% of the data variation and discriminated only samples of young shoots from different flush cycles, while all mature shoots from different seasons clustered together (Fig 3). PC2 was significantly correlated with six amino acids, positively with alanine, asparagine and tryptophan, and negatively with aspartic acid, glutamine and cystine. Thus, the amino acid profiles of phloem sap were more distinct between flush shoot growth stages than between seasons.

### *D*. *citri* population dynamics on young and mature flush stages of grapefruit and lemon

**Fig 4:**
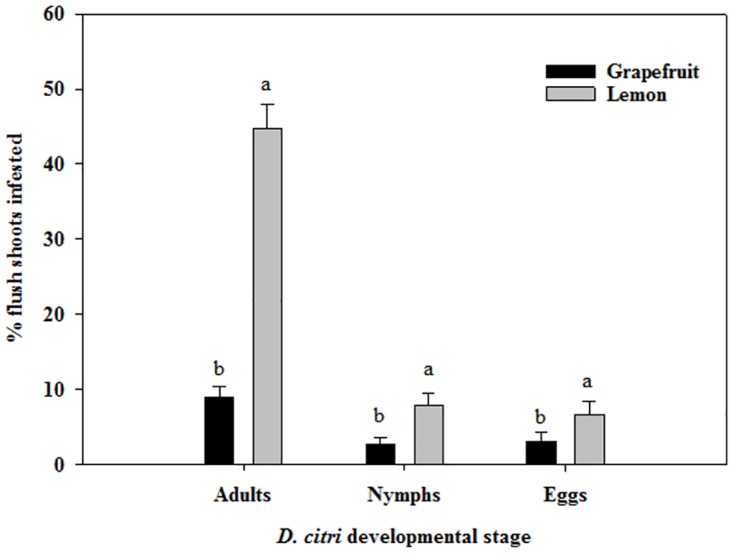
Relative abundances of different life stages of *Diaphorina citri* on flush shoots of Rio Red grapefruit and Meyer lemon (Weslaco, TX, 2014–2015). The experimental trees did not receive insecticidal application for the entire duration of the study.

**Fig 5:**
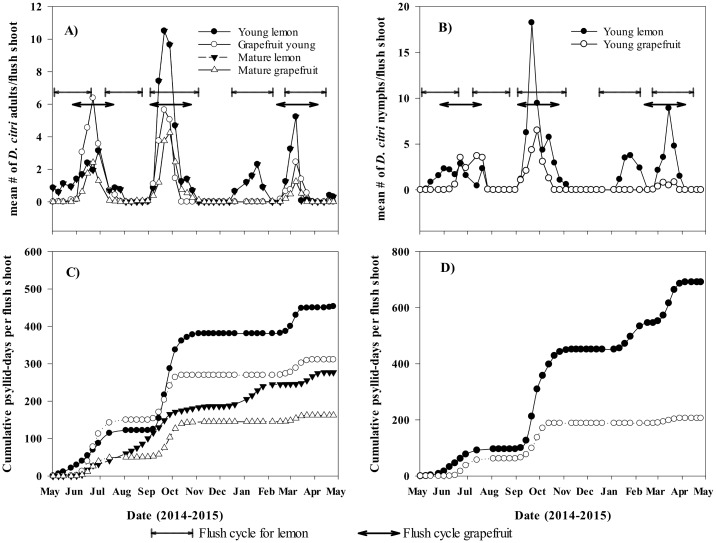
Densities of *Diaphorina citri* adults (A) and immatures (B) and corresponding cumulative-psyllid days (C and D) on young and mature ‘Rio Red’ grapefruit and ‘Meyer’ lemon; flush shoots (Weslaco, TX, 2014).

Densities of *D*. *citri* and the percentages of flush shoots infested by its life stages varied significantly (*P* < 0.05) with host plant, sampling time and the host plant by sampling time interaction (S2 Table). The exceptions were numbers of *D*. *citri* nymphs per flush shoot that were not affected by host plant and the percentage of flush shoots infested by *D*. *citri* eggs that were similar for both cultivars (S2 Table). Significantly more lemon flush shoots were infested by *D*. *citri* nymphs and adults compared to grapefruit flush shoots (Fig 4). Similarly, lemon flush shoots harbored significantly higher densities of *D*. *citri* adults than their grapefruit counterparts (Fig 5, S2 Table). The presence of *D*. *citri* life stages varied with time of the year. *D*. *citri* were mostly recovered during flush cycles when young expanding flush shoots were present. The highest densities of all *D*. *citri* life stages were recorded during the fall flush cycle as compared to the spring and summer flush cycles in agreement with Sétamou and Bartels [27]. An additional cycle was observed for lemon in winter (January), but significantly lower densities of *D*. *citri* were recorded during this winter flush cycle compared to others (Fig 5). Young flush shoots when present harbored significantly higher numbers of *D*. *citri* adults than mature flush shoots regardless of host plant (Fig 5, S2 Table). *D*. *citri* immatures were only recorded on the young expanding flush shoots. The observed significant interactions between sampling time, host plant and flush stages (S2 Table) mainly due to the non-synchrony of flush cycles for the two host plants evaluated, made it difficult to clearly discern *D*. *citri* populations among the two flush growth stages and the two host plants (Fig 5). To overcome this difficulty, cumulative psyllid-days were calculated for nymphs and adults recorded on young and mature shoots of both host plants (Fig 5). Cumulative psyllid-days remained constant and parallel to the *x*-axis when few or no psyllids were recovered from flush shoots during the sampling periods. Significantly higher cumulative psyllid-day values were obtained for *D*. *citri* nymphs (*χ*^2^ = 262.4, df = 1, *P* < 0.0001) and adults (*χ*^2^ = 26.5, df = 1, *P* < 0.0001) on young lemon shoots relative to their respective grapefruit counterparts. On mature shoots, cumulative adult psyllid-days were also significantly higher on lemon compared to grapefruit. In addition, for each host plant, cumulative adult psyllid-days were significantly higher on young compared to mature shoots (*χ*^2^ = 46.7, df = 1, *P* < 0.0001 for grapefruit and *χ*^2^ = 42.4 df = 1, *P* < 0.0001 for lemon).

### Feeding preferences of *D*. *citri*

**Fig 6:**
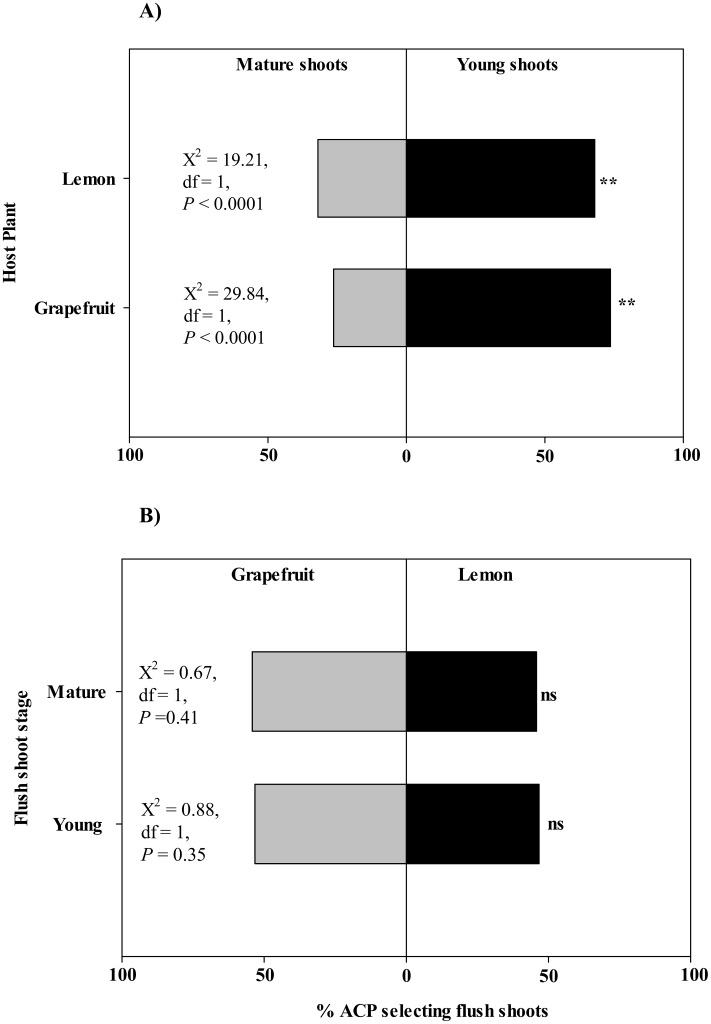
Settling preference of *Diaphorina citri* adults on young and mature flush shoots of grapefruit and lemon. Section A shows the preference between young and mature flush shoots of the same host plant species, while section B represents the selection of flush shoots of the same age between the two host plants.

A choice experiment was performed in the laboratory to test whether the higher colonization of young relative to mature flush shoots by *D*. *citri* adults and the differential densities of various life stages between lemon and grapefruit observed in the field were due to host preference. *D*. *citri* preference for young and mature shoots was evaluated separately for grapefruit and lemon. In addition, comparisons were made across host plant species for each growth stage. In choice tests between young and mature flush shoots, *D*. *citri* adults exhibited a strong preference for young shoots (Fig 6A). More than 68% of adults selected young flush shoots over mature ones for both grapefruit and lemon (χ^2^ ≥ 19.21, df = 1, *P* < 0.0001). For each flush shoot growth stage (young or mature), there were no significant differences in *D*. *citri* preference for grapefruit or lemon (Fig 6B). The majority (95.5%) of *D*. *citri* adults (95.5%) released in the choice arena were recorded feeding on young flush shoots of lemon and grapefruit. In contrast, only 48% of *D*. *citri* adults were recorded feeding on mature shoots of both host plants further confirming the preference of *D*. *citri* for young expanding shoots regardless of cultivar.

### Relationships between flush shoot characteristics and *D*. *citri*

The cumulative adult psyllid-days was negatively related to flush shoot hardness (*y* = 2.63–1.7*x*, df = 1, 2; R^2^ = 0.84), albeit marginally significant (*P* = 0.08) (S1 Fig). Correlation analyses revealed that cumulative adult psyllid-days were positively related to the nutrients N, P, C, and the N:C, N:S and N:Ca ratios of leaf tissue. In contrast, leaf Ca, Mn, S, and B levels were negatively and significantly related to cumulative adult psyllid-days (S3 Table). Notably, cumulative psyllid nymph-days were only affected by N:Ca ratio of leaf tissue (r = 0.83, *P* < 0.01, S3 Table), suggesting that over-fertilization of citrus with nitrogen without calcium uptake and/or supplementation may result in higher psyllid infestation and nymphal survival and development. The total free amino acids and the total essential amino acids were positively (*P* < 0.05) related to cumulative adult psyllid-days. With the exception of tryptophan that was not significantly related to cumulative adult psyllid-days, all other individual essential amino acids were positively correlated (*P* < 0.05) with this psyllid parameter (S3 Table).

## Discussion

An in-depth analysis revealed considerable differences in the physical and chemical characteristics of citrus flush shoot growth stages. Young flush shoots were softer with lower Ca concentrations compared to matured flush tissues (Table 1). Calcium is a key component of plant cell walls and thereby plays a vital role in the structural integrity of plant tissues [38,39]. Young flush shoots lack well-developed cell walls that provide structural integrity and constitute the first line of host plant defense against pests and pathogens [40]. Such shoots are therefore more attractive for feeding and egg-laying by *D*. *citri* since they are probably easier to penetrate as indicated by the softness data. During oviposition *D*. *citri* eggs are laid inserted with a stalk into leaf petiole [28]. Exogenous application of calcium to developing shoots has been associated with reduced *D*. *citri* population and infestation levels [41,42] presumably due to enhanced cell wall thickening and lignification which are unsuitable for egg-laying or feeding. Taken together, *D*. *citri* preference for oviposition on younger and softer flush shoot stages may be due in part to the fact that such tissues are still developing in contrast to the Ca-dependent structural integrity and physical barrier to penetration that mature shoots already possess.

Nitrogen is critical for amino acid and protein formation. Higher levels of N and essential amino acids are important for the development and reproduction of phloem feeding insects [16,19], such as *D*. *citri*. Low N content and low N:C ratio are widely recognized as indicators of low plant nutritional quality for sap sucking insects [16]. Young flush shoots had higher levels of critical building blocks of free amino acids including N, P and C and higher N:C ratios than mature shoots (Table 1). Phloem sap of young flush shoots also contained higher amounts of total and essential amino acids (Table 2, S1 Table). This is consistent with the fact that young developing shoots are essentially sinks for carbohydrates and other essential substrates required for development [43]. The greater abundance of these nutritional compounds in young shoots makes them a preferred tissue for feeding and reproduction. This is consistent with the observed higher densities of *D*. *citri* life stages on young shoots in field studies (Fig 5) and the preference of the same growth stage by adult psyllids in the choice experiment (Fig 6). The cumulative adult psyllid-days, an indication of the psyllid density and residence time on flush shoots was strongly correlated with N, Ca, total free amino acids (TAA) and essential free amino acid (EAA) concentrations in shoot tissues (S3 Table). The correlations were positive for N, TAA and EAA (0.66, 0.56 and 0.59, respectively) and negative for Ca (-0.74) (S3 Table), indicating that adult psyllids had a strong preference for young tender, nutritious tissues with high protein substrates than the high-Ca, lignified mature shoots. The relative abundance of key nutrients in phloem sap of young flush shoots relative to mature ones is not surprising. From emergence to expansion, the young shoot is a sink tissue and net importer of substrates, especially N and C, almost entirely via the phloem [43]. As flush shoot matures and leaf development and expansion continue, young shoots change from sinks to sources [43], thus explaining the depletion of many nutrients and reduced concentrations of amino acids observed in mature shoots relative to young ones. This transition from nutrient sink to source significantly affects the C and N economy of the leaf. [43]. Thus, the decrease in N:C and N:Ca ratios observed with flush shoot maturity may be a consequence of ontogenesis as newly formed leaves change from physiological assimilate sinks to sources.

The roles of essential amino acids in the performance and abundance of phloem-feeding insects have been well elucidated [19]. The results obtained in this study indicated that all but one essential amino acid (tryptophan) were significantly and positively related to adult psyllid load during the season (S3 Table) suggesting their role in host selection and performance. As shown in Table 2, the concentrations of total and essential amino acids were several-fold higher in young versus mature flush shoot stages regardless of host plant or season. This observation is consistent with the tissue preference data indicating that young flush shoots are the preferred sites for psyllid oviposition and nymphal development. It is plausible that *D*. *citr*i has co-evolved with its rutaceous host species to precisely time its mating, oviposition, egg maturation and immature development to coincide with the onset of new flush production. In general, higher psyllid densities were observed during each flush cycle on both host plants (Fig 5). Although total and essential amino acid concentrations were higher in phloem sap during the spring relative to the fall flush cycle (Table 2), this was not accompanied by concomitant higher psyllid densities in spring as shown in Fig 5. A possible explanation for this disparity could be that the spring flush, though nutritionally richer than fall flushes, follows a long psyllid overwintering period—from November to February under South Texas conditions [24]. Therefore, fewer adult psyllids would be available to initiate reproduction during the spring relative to more abundant adults in the fall. Nonetheless, the nutritional content of young expanding shoots seemed sufficient enough to sustain successful reproduction of *D*. *citri* relative to mature flush shoots at any given flush cycle season.

Results of the *D*. *citri* feeding preference study clearly demonstrated that flush shoot growth stage had a stronger influence on psyllid substrate selection than host plant species (Fig 6). In previous studies, *D*. *citri* population densities have been reported to vary with host plants [24,44]. Under field conditions in Texas, for instance, some rutaceous hosts such as lemon tend to have more flushes per year than others such as grapefruit. In this study, we did not observe a preferential selection between grapefruit and lemon for either young or mature shoots. Therefore the observed variation in host-specific psyllid densities under field conditions may be attributed to the frequency and availability of flush shoots rather than host preference.

In conclusion, the results of this study highlight the importance of young flush shoots in the population dynamics of the Asian citrus psyllid with consequences for vector management. The data indicate that both physical characteristics and nutritional composition of flush shoots and their phloem sap are important factors regulating host colonization and reproductive potential of *D*. *citri* in citrus groves. Therefore, with regards to psyllid abundance in the environment, flush shoot quality in terms of softness and nutritional content matters.

## Supporting Information

S1 Fig Relationship between flush shoot hardness (kgf) and the cumulative adult-psyllid days on grapefruit and lemon. (PDF)

S1 Table Seasonal mean concentration of amino acids (μg ml^-1^ phloem fluid) detected in phloem sap of young and mature flush shoots of grapefruit and lemon trees. (PDF)

S2 Table Linear mixed model analysis of variance of factors affecting densities and flush shoot infestation of *Diaphorina citri* on grapefruit and lemon. (PDF)

S3 Table Matrix of correlation coefficients between various leaf mineral content, phloem sap amino acids, cumulative psyllid-days (CAD) and cumulative psyllid nymph-days (CND) recorded on young and mature flush shoots of grapefruit and lemon trees (Weslaco, TX 2014–2015). (PDF)
