# Supplementary figures and images for: Quality Matters: Influences of Citrus Flush Physicochemical Characteristics on Population Dynamics of the Asian Citrus Psyllid (Hemiptera: Liviidae)
Source: PLoS One. 2016 Dec 28;11(12):e0168997. doi: 10.1371/journal.pone.0168997 (PMC5193449; doi:10.1371/journal.pone.0168997)

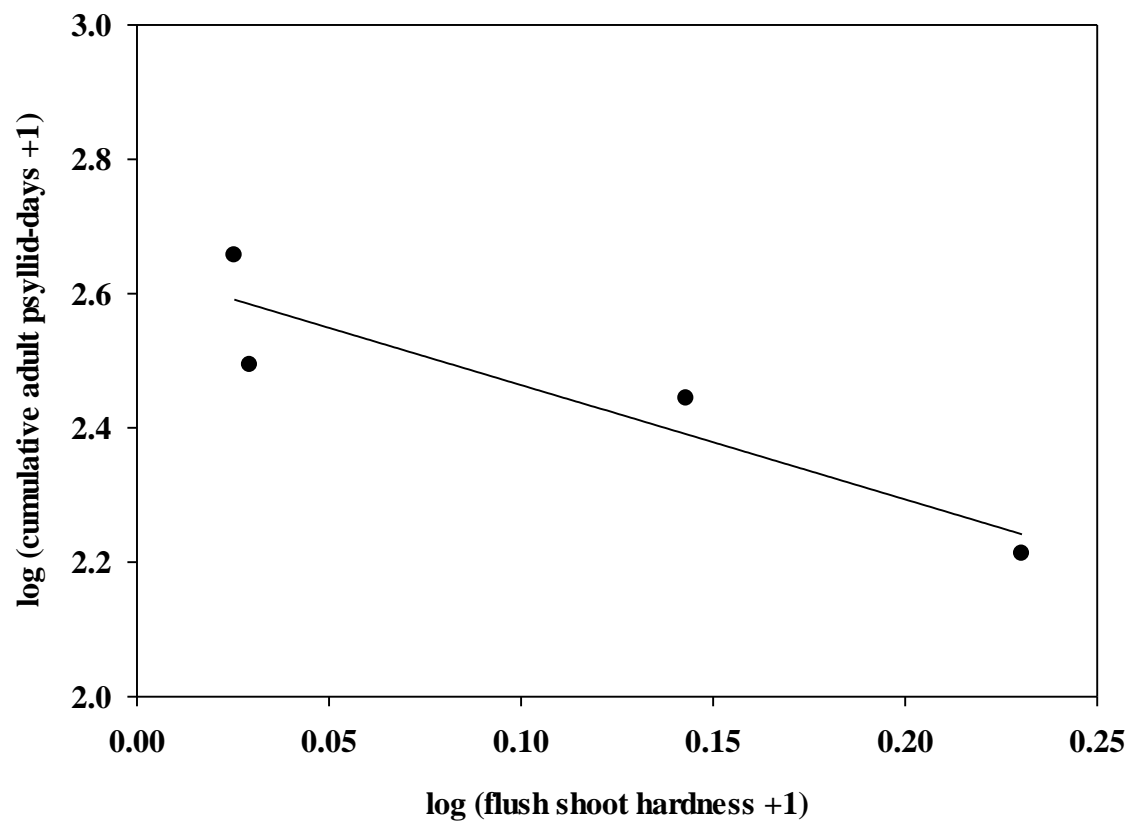

Supplement: S1 Fig — (PDF) [file pone.0168997.s001.pdf]
