# Supplementary material for: Quality Matters: Influences of Citrus Flush Physicochemical Characteristics on Population Dynamics of the Asian Citrus Psyllid (Hemiptera: Liviidae)
Source: PLoS One. 2016 Dec 28;11(12):e0168997. doi: 10.1371/journal.pone.0168997 (PMC5193449; doi:10.1371/journal.pone.0168997)
Supplement: S1 Table — (PDF) [file pone.0168997.s002.pdf]

S1 Table . Seasonal mean concentration of amino acids (μg ml^{-1} phloem fluid) detected in phloem sap of young and mature flush shoots of grapefruit and lemon trees

	Grapefruit								Lemon							
	Jul-14		Sep-14		Dec-14*		Mar-15		Jul-14		Sep-14		Dec-14*		Mar-15	
μg L^{-1}	Y	M	Y	M	Y	M	Y	M	Y	M	Y	M	Y	M	Y	M
1-methyl-histidine	8.58	0.00	9.17	0.00	-	0.00	17.61	0.00	11.22	0.00	3.69	0.00	-	0.00	6.68	0.00
3-methyl-histidine	0.20	0.00	0.19	0.00	-	0.00	1.56	0.00	0.90	0.00	0.00	0.00	-	0.00	0.29	0.00
Alanine	57.20	1.93	51.33	2.89	-	3.87	261.82	1.92	64.96	3.27	71.37	3.14	-	3.74	159.68	5.74
Arginine	115.26	5.56	23.62	3.19	-	14.93	64.65	7.24	43.55	4.89	23.85	6.32	-	4.38	40.90	8.55
Asparagine	191.13	16.13	129.68	15.94	-	9.41	415.75	41.16	52.54	21.08	164.24	19.94	-	21.31	580.70	29.62
Aspartic Acid	19.21	0.79	7.67	2.02	-	1.17	7.55	2.46	25.98	1.19	5.36	1.51	-	0.81	9.54	3.44
Citrulline	2.52	0.08	0.68	0.43	-	0.00	0.00	0.00	2.03	1.73	0.00	0.47	-	0.05	0.00	0.00
Cystathionine/allocystathionine	0.00	0.00	0.00	0.44	-	0.13	0.00	0.00	0.00	0.16	0.00	0.42	-	0.14	0.00	0.00
Cystine	2.09	0.00	0.30	0.00	-	0.00	2.12	0.00	2.43	0.00	0.54	0.00	-	0.00	0.00	0.00
Ethanolamine	12.53	0.20	5.97	0.84	-	0.69	26.51	1.07	20.75	0.26	4.03	0.71	-	0.40	12.61	1.55
Glutamic Acid	17.27	2.51	31.61	2.73	-	4.21	18.26	2.28	31.72	4.46	21.45	4.54	-	4.85	26.61	7.10
Glutamine	80.13	3.21	35.33	2.39	-	2.57	42.53	3.78	40.76	5.94	13.98	3.53	-	1.75	23.88	6.06
Glycine	17.89	1.88	12.15	3.03	-	4.74	39.65	1.28	20.13	4.09	16.26	2.50	-	5.27	29.46	2.00
Histidine	12.45	0.95	7.98	1.59	-	1.84	19.92	1.39	15.14	1.87	6.92	0.86	-	1.64	12.87	0.85
Hydroxylysine	0.30	0.00	0.00	0.00	-	0.00	1.71	0.00	1.50	0.00	0.00	0.00	-	0.00	0.43	0.00
Isoleucine	12.81	0.36	9.57	0.59	-	0.45	26.40	0.00	17.49	0.56	6.26	0.41	-	0.40	13.45	0.62
Leucine	22.88	0.83	15.08	0.72	-	1.25	53.96	0.67	44.94	1.08	12.07	0.78	-	1.05	24.87	1.51
Lysine	45.03	1.38	29.16	1.94	-	2.11	47.51	1.34	49.56	1.22	16.48	1.58	-	1.36	23.74	1.56
Methionine	4.24	0.11	2.97	0.22	-	0.07	9.97	0.16	9.42	0.09	2.30	0.16	-	0.00	5.14	0.18
Ornithine	6.19	1.21	3.25	1.79	-	3.99	3.12	0.00	4.74	2.87	4.16	1.27	-	3.89	2.21	0.20

Phenylalanine	30.62	1.09	13.77	0.92	-	0.82	28.99	0.95	33.65	1.19	8.59	0.56	-	0.64	18.03	1.36
Phosphoethanolamine	13.75	7.81	9.58	7.40	-	16.04	24.14	9.50	6.02	11.25	8.46	13.97	-	15.47	15.40	11.49
Phosphoserine	15.20	6.51	19.23	6.10	-	6.52	18.94	4.69	28.44	8.26	10.40	6.19	-	6.25	11.06	7.35
Proline	575.78	23.03	299.85	36.90	-	91.19	998.51	39.34	422.19	32.37	347.69	36.73	-	70.17	840.68	58.94
Sarcosine	5.80	0.00	8.30	0.12	-	0.00	27.76	0.60	8.37	0.00	4.27	0.00	-	0.00	9.83	0.84
Serine	155.91	7.78	67.70	12.63	-	7.03	199.33	4.77	148.67	11.46	44.74	5.60	-	6.36	143.83	11.09
Taurine	0.00	0.00	2.29	0.00	-	0.44	11.83	0.51	0.00	0.00	1.27	0.00	-	0.35	5.25	0.00
Threonine	31.46	1.75	11.77	1.62	-	8.46	58.48	1.48	34.43	2.47	28.40	7.35	-	8.79	39.41	2.76
Tryptophan	0.00	0.00	4.30	0.00	-	0.00	46.27	0.00	12.42	0.00	0.00	0.00	-	0.00	31.53	0.00
Tyrosine	49.76	1.05	30.57	1.60	-	2.19	56.19	0.73	72.17	1.47	18.69	1.36	-	1.96	31.61	1.22
Valine	28.19	1.36	20.96	1.56	-	1.31	49.48	1.04	34.62	1.59	13.31	1.16	-	1.13	29.52	2.67
α-amino-adipic acid	12.46	0.10	16.11	0.10	-	0.37	38.15	0.51	9.48	0.00	2.20	0.00	-	0.00	9.42	0.45
α-amino-n-butyric acid	2.70	0.00	4.55	2.74	-	0.00	4.75	0.13	4.85	0.00	2.39	2.42	-	0.00	2.19	0.38
β-alanine	10.46	0.00	8.47	0.00	-	0.00	20.36	0.00	7.52	0.00	3.08	0.00	-	0.00	6.88	0.00
β-amino-isobutyric acid	0.05	0.00	0.00	0.00	-	0.00	1.85	0.00	1.73	0.00	0.00	0.00	-	0.00	21.79	0.00
γ-amino-butyric acid	208.99	5.60	107.39	9.28	-	12.43	379.45	4.53	191.45	4.71	105.28	6.99	-	5.72	48.48	4.17

*Young flush shoots not present at this time.
