# Supplementary material for: Quality Matters: Influences of Citrus Flush Physicochemical Characteristics on Population Dynamics of the Asian Citrus Psyllid (Hemiptera: Liviidae)
Source: PLoS One. 2016 Dec 28;11(12):e0168997. doi: 10.1371/journal.pone.0168997 (PMC5193449; doi:10.1371/journal.pone.0168997)
Supplement: S2 Table — (PDF) [file pone.0168997.s003.pdf]

S2 Table . Linear mixed model analysis of variance of factors affecting densities and flush shoot infestation of *Diaphorina citri* on grapefruit and lemon.

	***F*-values for *D. citri* densities per flush**					
**Source of variation**	**Adults**		**Nymphs**		**Eggs**	
	df	*F*	df	*F*	df	*F*
Host plant (Host)	1,152	29.08**	1,152	0.44[ns]	1,152	31.15**
Flush growth stage (Stage)	1,152	22.80**	1,152	10.05**	1,152	27.42**
Sampling date (Time)	23,952	40.08**	23,952	51.66**	23,952	58.08**
Host × Time	23,952	24.37**	23,952	38.70**	23,952	45.69**
Stage × Time	23,952	6.29**	23,952	2.75**	23,952	5.55**
Host × Stage	1,952	10.42**	1,952	14.70**	1,952	29.53**
	***F*-values for *D. citri* flush shoot infestation level**					
	**Adults**		**Nymphs**		**Eggs**	
	df	*F*	df	*F*	df	*F*
Host plant (Host)	1,23	28.13**	1,23	5.95*	1,23	4.33[ns]
Sampling date (Time)	23,198	6.10**	23,198	6.55**	23,198	6.55**
Host × Time	23,198	6.14**	23,198	2.56**	23,198	2.86**

[1] ns= not signif
