# Supplementary material for: Quality Matters: Influences of Citrus Flush Physicochemical Characteristics on Population Dynamics of the Asian Citrus Psyllid (Hemiptera: Liviidae)
Source: PLoS One. 2016 Dec 28;11(12):e0168997. doi: 10.1371/journal.pone.0168997 (PMC5193449; doi:10.1371/journal.pone.0168997)
Supplement: S3 Table — (PDF) [file pone.0168997.s004.pdf]

S3 Table . Matrix of correlation coefficients between various leaf mineral content, phloem sap amino acids, cumulative psyllid-days (CAD) and cumulative psyllid nymph-days (CND) recorded on young and mature flush shoots of grapefruit and lemon trees (Weslaco, TX 2014-2015).

	CAD	CND	N	Ph	K	Ca	Mg	Na	Zn	Fe	Cu	Mn	S	B	C	N:C	N:S	N:Ca	Ala	Arg	Asn	Asp	Cys	Glu	Gln	Gly	His	Ile	Leu	Lys	Met	Phe	Pro	Ser	Thr	Prp	Tyr	Val	FAA	EAA	GluGln
CAD	1.00																																							
CND	0.61	1.00																																						
N	**0.66**	0.66	1.00																																					
P	**0.62**	0.43	**0.93**	1.00																																				
K	0.48	0.36	**0.83**	**0.88**	1.00																																			
Ca	**-0.74**	-0.39	**-0.82**	**-0.89**	**-0.86**	1.00																																		
Mg	-0.39	0.62	-0.49	-0.49	-0.31	**0.57**	1.00																																	
Na	0.51	0.14	**0.72**	**0.65**	**0.62**	**-0.74**	**-0.64**	1.00																																
Zn	0.49	0.59	**0.91**	**0.90**	**0.75**	**-0.71**	-0.38	**0.68**	1.00																															
Fe	-0.06	0.32	-0.31	-0.47	**-0.62**	0.43	-0.06	-0.49	-0.37	1.00																														
Cu	0.43	0.55	0.47	0.53	0.44	-0.49	-0.21	0.20	**0.55**	0.01	1.00																													
Mn	**-0.66**	0.66	-0.53	**-0.68**	**-0.66**	**0.87**	0.52	-0.52	-0.38	0.32	**-0.55**	1.00																												
S	**-0.58**	0.34	-0.29	-0.40	-0.37	**0.71**	**0.56**	**-0.62**	-0.24	0.18	-0.43	**0.81**	1.00																											
B	**-0.74**	0.29	**-0.77**	**-0.76**	**-0.59**	**0.85**	**0.68**	**-0.68**	**-0.62**	-0.04	**-0.54**	**0.78**	**0.61**	1.00																										
C	**0.66**	-0.40	**0.82**	**0.93**	**0.80**	**-0.92**	**-0.57**	**0.62**	**0.74**	-0.40	0.37	**-0.78**	-0.53	**-0.83**	1.00																									
N:C	**0.56**	0.59	**0.84**	**0.80**	**0.65**	**-0.59**	-0.40	0.43	**0.79**	-0.20	0.53	-0.35	-0.16	**-0.57**	**0.60**	1.00																								
N:S	**0.78**	0.70	**0.95**	**0.94**	**0.83**	**-0.94**	**-0.61**	**0.78**	**0.85**	-0.31	0.53	**-0.73**	**-0.56**	**-0.87**	**0.90**	**0.78**	1.00																							
N:Ca	**0.79**	**0.83**	**0.92**	**0.93**	**0.85**	**-0.92**	-0.50	**0.71**	**0.79**	-0.43	0.40	**-0.68**	-0.49	**-0.78**	**0.89**	**0.77**	**0.96**	1.00																						
Ala	0.50	-0.23	**0.75**	**0.81**	**0.61**	**-0.69**	-0.53	**0.66**	**0.73**	-0.34	0.38	**-0.58**	-0.36	**-0.62**	**0.79**	**0.65**	**0.77**	**0.67**	1.00																					
Arg	0.46	-0.60	0.53	0.60	**0.63**	**-0.71**	-0.52	**0.57**	0.27	-0.40	-0.05	**-0.67**	-0.44	**-0.59**	**0.70**	0.37	**0.62**	**0.68**	0.57	1.00																				
Asn	0.33	-0.11	**0.77**	**0.83**	**0.67**	**-0.66**	-0.51	**0.71**	**0.79**	-0.38	0.42	-0.49	-0.30	**-0.59**	**0.71**	**0.78**	**0.75**	**0.66**	**0.88**	0.54	1.00																			
Asp	0.37	-0.52	0.51	0.50	**0.62**	**-0.71**	-0.40	**0.68**	0.32	-0.37	-0.08	**-0.58**	-0.40	**-0.58**	**0.65**	0.06	**0.58**	**0.59**	0.36	**0.74**	0.30	1.00																		
Cys	**0.56**	-0.54	0.48	0.49	0.52	**-0.66**	-0.43	0.55	0.24	-0.31	-0.08	**-0.63**	-0.41	**-0.57**	**0.67**	0.10	**0.58**	**0.58**	**0.56**	**0.81**	0.30	**0.85**	1.00																	
Glu	0.51	-0.03	**0.78**	**0.85**	**0.79**	**-0.87**	-0.48	**0.64**	**0.76**	-0.40	0.33	**-0.65**	-0.45	**-0.75**	**0.91**	0.44	**0.82**	**0.81**	**0.58**	0.52	**0.57**	**0.74**	**0.57**	1.00																	
Gln	0.48	-0.73	0.51	**0.61**	**0.65**	**-0.76**	-0.51	0.54	0.26	-0.39	-0.03	**-0.75**	-0.49	**-0.64**	**0.78**	0.28	**0.63**	**0.69**	0.52	**0.96**	**0.46**	**0.82**	**0.83**	**0.65**	1.00																
Gly	0.53	-0.32	**0.83**	**0.88**	**0.73**	**-0.78**	**-0.55**	**0.72**	**0.77**	-0.41	0.33	**-0.62**	-0.35	**-0.70**	**0.87**	**0.65**	**0.84**	**0.77**	**0.97**	**0.69**	**0.87**	**0.55**	**0.68**	**0.71**	**0.65**	1.00															
His	0.54	-0.66	**0.77**	**0.83**	**0.75**	**-0.83**	-0.54	**0.74**	**0.66**	-0.44	0.23	**-0.71**	-0.43	**-0.73**	**0.90**	0.47	**0.82**	**0.77**	**0.88**	**0.78**	**0.74**	**0.75**	**0.84**	**0.78**	**0.80**	**0.96**	1.00														
Ile	**0.55**	-0.64	**0.73**	**0.80**	**0.70**	**-0.80**	**-0.55**	**0.71**	**0.62**	-0.39	0.24	**-0.72**	-0.45	**-0.71**	**0.88**	0.44	**0.79**	**0.72**	**0.90**	**0.74**	**0.72**	**0.70**	**0.83**	**0.75**	**0.76**	**0.95**	**0.99**	1.00													
Leu	**0.56**	-0.57	**0.69**	**0.73**	**0.65**	**-0.76**	-0.52	**0.72**	**0.59**	-0.36	0.18	**-0.66**	-0.41	**-0.67**	**0.83**	0.34	**0.75**	**0.67**	**0.86**	**0.69**	**0.64**	**0.74**	**0.87**	**0.73**	**0.72**	**0.92**	**0.98**	**0.99**	1.00												
Lys	**0.58**	-0.82	**0.67**	**0.75**	**0.73**	**-0.85**	-0.53	**0.67**	0.50	-0.41	0.13	**-0.77**	-0.50	**-0.73**	**0.89**	0.32	**0.77**	**0.75**	**0.71**	**0.84**	**0.56**	**0.87**	**0.92**	**0.81**	**0.90**	**0.83**	**0.95**	**0.94**	**0.93**	1.00											
Met	**0.56**	-0.57	**0.70**	**0.72**	**0.66**	**-0.77**	-0.50	**0.74**	**0.60**	-0.36	0.19	**-0.66**	-0.42	**-0.68**	0.82	0.32	**0.75**	**0.66**	**0.83**	**0.67**	**0.63**	**0.77**	**0.86**	**0.76**	**0.71**	**0.90**	**0.97**	**0.98**	**1.00**	**0.93**	1.00										
Phen	**0.57**	-0.80	**0.67**	**0.71**	**0.72**	**-0.82**	-0.53	**0.73**	0.48	-0.41	0.07	**-0.72**	-0.46	**-0.71**	**0.84**	0.30	**0.74**	**0.72**	**0.70**	**0.86**	**0.57**	**0.90**	**0.92**	**0.78**	**0.90**	**0.83**	**0.95**	**0.93**	**0.93**	**0.99**	**0.93**	1.00									
Pro	0.49	-0.41	**0.81**	**0.87**	**0.74**	**-0.79**	**-0.58**	**0.75**	**0.74**	-0.43	0.32	**-0.65**	-0.40	**-0.70**	**0.86**	**0.66**	**0.83**	**0.77**	**0.95**	**0.75**	**0.91**	**0.56**	**0.65**	**0.69**	**0.71**	**0.98**	**0.94**	**0.93**	**0.88**	**0.82**	**0.86**	**0.83**	1.00								
Ser	0.49	-0.77	**0.73**	**0.79**	**0.73**	**-0.81**	-0.56	**0.77**	**0.58**	-0.42	0.16	**-0.71**	-0.43	**-0.71**	**0.86**	0.45	**0.78**	**0.73**	**0.85**	**0.85**	**0.76**	**0.77**	**0.84**	**0.74**	**0.85**	**0.93**	**0.99**	**0.97**	**0.95**	**0.95**	**0.94**	**0.96**	**0.94**	1.00							
Thr	**0.58**	-0.27	**0.84**	**0.85**	**0.72**	**-0.79**	**-0.60**	**0.74**	**0.73**	-0.38	0.30	**-0.63**	-0.39	**-0.73**	**0.84**	**0.62**	**0.86**	**0.79**	**0.93**	**0.74**	**0.81**	**0.62**	**0.76**	**0.69**	**0.69**	**0.98**	**0.96**	**0.95**	**0.93**	**0.85**	**0.91**	**0.86**	**0.96**	**0.94**	1.00						
Trp	0.28	-0.35	**0.61**	**0.67**	0.48	-0.52	-0.45	**0.66**	**0.65**	-0.27	0.33	-0.44	-0.25	-0.50	**0.64**	0.49	**0.61**	0.45	**0.95**	0.41	**0.84**	0.29	0.46	0.48	0.37	**0.90**	**0.80**	**0.83**	**0.81**	**0.60**	**0.79**	**0.61**	**0.88**	**0.78**	**0.84**	1.00					
Tyr	0.51	-0.73	**0.67**	**0.71**	**0.72**	**-0.82**	-0.50	**0.70**	0.51	-0.40	0.10	**-0.71**	-0.45	**-0.71**	**0.85**	0.26	**0.74**	**0.71**	**0.69**	**0.78**	0.53	**0.90**	**0.92**	**0.82**	**0.85**	**0.82**	**0.95**	**0.93**	**0.95**	**0.99**	**0.95**	**0.99**	**0.80**	**0.94**	**0.85**	**0.61**	1.00				
Val	**0.56**	-0.71	**0.75**	**0.82**	**0.73**	**-0.83**	**-0.57**	**0.74**	**0.64**	-0.41	0.24	**-0.73**	-0.47	**-0.73**	**0.90**	0.46	**0.81**	**0.75**	**0.89**	**0.77**	**0.74**	**0.73**	**0.82**	**0.78**	**0.79**	**0.95**	**1.00**	**1.00**	**0.98**	**0.95**	**0.97**	**0.94**	**0.94**	**0.98**	**0.94**	**0.82**	**0.94**	1.00			
FAA	**0.56**	-0.52	**0.79**	**0.86**	**0.74**	**-0.81**	**-0.59**	**0.75**	**0.70**	-0.42	0.29	**-0.69**	-0.43	**-0.72**	**0.88**	**0.61**	**0.84**	**0.77**	**0.95**	**0.78**	**0.87**	**0.61**	**0.72**	**0.72**	**0.75**	**0.99**	**0.97**	**0.96**	**0.92**	**0.87**	**0.90**	**0.87**	**0.99**	**0.97**	**0.97**	**0.87**	**0.85**	**0.97**	1.00		
EAA	**0.59**	-0.73	**0.73**	**0.79**	**0.73**	**-0.82**	**-0.58**	**0.74**	**0.58**	-0.42	0.17	**-0.73**	-0.46	**-0.72**	**0.88**	0.45	**0.80**	**0.75**	**0.85**	**0.85**	**0.72**	**0.77**	**0.87**	**0.74**	**0.85**	**0.93**	**0.99**	**0.98**	**0.96**	**0.96**	**0.95**	**0.97**	**0.93**	**0.99**	**0.95**	**0.76**	**0.95**	**0.99**	**0.96**	1.00	
Glu + Gln	**0.55**	**-0.87**	**0.65**	**0.75**	**0.75**	**-0.87**	**-0.54**	**0.62**	0.46	-0.43	0.10	**-0.78**	-0.52	**-0.74**	**0.89**	0.36	**0.75**	**0.79**	**0.59**	**0.89**	**0.54**	**0.86**	**0.81**	**0.83**	**0.96**	**0.73**	**0.86**	**0.83**	**0.78**	**0.95**	**0.79**	**0.94**	**0.76**	**0.88**	**0.75**	0.44	**0.91**	**0.86**	**0.80**	**0.88**	1.00

Correlation coefficients in bold fonts are significant (P< 0.05).
